# Grit and Resilience as Predictors of Creativity Among Chinese English as a Foreign Language Teachers

**DOI:** 10.3389/fpsyg.2022.923313

**Published:** 2022-06-21

**Authors:** Jia Sun

**Affiliations:** Department of Foreign Languages, Fuzhou University Zhicheng College, Fuzhou, China

**Keywords:** Chinese EFL teachers, creativity, grit, resilience, persistence, individual skills

## Abstract

Teachers have been viewed for many years as one of the most effective factors with an important role in academic and learning settings. Numerous studies have been carried out on teachers and their performances in the classroom. Feelings are one of the pillars of all humans which can have a crucial function in offering academia that can impact all domains of learning. Creativity is one of the subcategories of feelings that is worthy to people and the community. Nonetheless, as a significant mental attribute, it has not been attended to enough by experts in language teaching until now. Some factors that seem concerning creativity are grit and resilience, the grit has a basic function in the educational and teaching cycle because gritty educators are more inspired to handle difficulties in hard situations. Moreover, to beware of these difficulties as a response to unprecedented situations, a similar intellectual concept rises in positive psychology known as resilience, which explains the persistence and highlights individuals’ skills. Therefore, the present study delineates the relationship of these notions with language teachers’ creativity. To this end, through convenient sampling 264 male and female Chinese EFL teachers took part in the present study, and their creativity, grit, and resilience were scrutinized by filling out the related questionnaires. The results through correlation coefficients indicated that creativity was negatively but significantly related to grit, but it was positively and significantly related to resilience. The results of the multiple regression showed that both grit and resilience could significantly predict creativity although grit is a better predictor of creativity. Some educational implications of the research about the outcomes of the research under academic circumstances are suggested.

## Introduction

Recently, there was an increasing interest in implementing Positive Psychology (PP) in the domain of language education among both learners and teachers (Dewaele et al., 2019). Positive psychology in language education alludes to an endeavor to learn the second or foreign language attainment from a more constructive viewpoint (MacIntyre et al., 2016; Wang et al., 2021). Under the structure of PP, various themes have been investigated, like feelings, flow, aspiration, creativity, and inquisitiveness (Lake, 2013; Dewaele and Dewaele, 2017). In academic contexts, creativity portrays how an individual thinks, learns, and creates information in school classes, like science and mathematics, which mirrors the attribute of problem-solving (Fan et al., 2021). Also, it is deemed as a personal factor that has been mainly unattended in second language attainment studies, aside from new endeavors (Albert and Kormos, 2004) and it is a vital component of education and creative education in which educators imagine, layout, and employ innovative education tendencies, approaches, or tasks to conform to learners’ intellectual improvement and provoke learners’ incentive to achieve the highest effect (Beaird et al., 2018). As a requirement to prosper in the 21st century, among different elements, educators’ creativity has been emphasized in schools and colleges, which hold the responsibility to assist with improving learners’ creativity, as well (Parker-Bell, 2010). Moreover, there is an increasing amount of proof connected to the health advantages of engaging in creative exercises, and involvement in creative encounters about the growth and sustainability of individual well-being (Dolan and Metcalfe, 2012; Conner et al., 2018). Some researchers (Ozkal, 2014; Gu, 2018; Huang et al., 2019) indicated that educators’ creativity alludes to the tactics employed as a branch of innovative and precious education guided by specific education ideas to improve learners’ learning interest to gain education purposes in EFL context. The core of creative education is to design and employ innovative, authentic, or original education approaches (Khurshid et al., 2012).

Furthermore, creativity mirrors numerous traits like taking risks, having a constructive function in creative thinking, articulating and characterizing issues, development and growth to conquer issues, tolerance for dual issues (ambiguity), and regard for others and the surrounding (Starko, 2013). For Metzl and Morrell (2008), creativity can be regarded as a character attribute of resilient individuals; thus, it has a basic function in the conceptualization of resilience as a multi-faceted cycle. Indeed, some experiential research has attended to what results in promotions of L2 teachers’ strong points and characteristics like resilience, joy, and positivity (MacIntyre and Mercer, 2014; Oxford, 2016). As opposed to the deconstructive aspects of instruction, PP encourages the practitioners to emphasize the strength of constructive feelings like happiness, interest, fervor, resilience, positivism, and the possibility to avoid deconstructive inconveniences (MacIntyre et al., 2019). Resilience is considered a significant part of promoting creativity even in the EFL context and among Chinese teachers and it is best portrayed in the PP pattern, which accentuates how individuals flourish and live more happily (MacIntyre et al., 2019; Wang et al., 2022). Educators’ resilience refers to the fact that they can adjust to diverse conditions thru adaptation and improve their capability to encounter destructive situations (Deng et al., 2020). In addition, cognitive interest, great degrees of devotion, self-esteem, emotive engagement, and attraction to intricacy and contrast are several primary character attributes that resilient and creative individuals might have in common.

Likewise, in any educational cycle, creativity demands teachers to be present and concentrate, thereby trying for grit (Yeh et al., 2019). Grit is the zeal that enhances numerous significant abilities like cooperation, creative thinking, and dealing with alterations that pave the path to a successful life (Roberts, 2009). The notion of grit, initially coined by Duckworth et al. (2007), has grown and extended in line with the domain of PP. It is a character attribute that is crucial to individual achievement and presentation across different educational and non-educational areas. It has been characterized as a compound attribute defined by persistence and enthusiasm for long-run objectives (Duckworth et al., 2007; Credé et al., 2017). As a non-cognitive different characteristic that affects language education outcomes, grit has also a significant role in personal fulfillment and affective paradigms such as unhappiness and passion (Steinmayr et al., 2018; Datu et al., 2019). Dweck (2009) remarks that grit and endeavor are the symbols of numerous creative geniuses who were simply normal individuals who became extremely encouraged with the skill to prosper on struggles. Dweck (2009) claims many creative scholars maintain that the impactful elements in creative success are persistence and resilience, which are the two primary attributes of grit. Due to the substantial advantages of creativity, it is significant to comprehend how creativity can be promoted and inspired. In the domain of second or foreign language education; nonetheless, creativity has almost been understudied (Dörnyei, 2005) and more or less unattended to (Albert, 2006). To the researchers’ knowledge, there is not enough research that takes into consideration the function of creativity in language educational success (Albert and Kormos, 2004; Sutrisno, 2007). Based on the above-mentioned studies on the role of grit and resilience among language teachers, to the best of the researcher’s knowledge, no research so far has been carried out on the role of these types of issues on teachers’ creativity. Subsequently, the following research is going to answer the subsequent questions:

RQ1: Does Chinese EFL teachers’ resilience significantly predict their creativity?

RQ2: Does Chinese EFL teachers’ grit significantly predict their creativity?

RQ3: Which one of the two variables, Chinese EFL teachers’ resilience or grit, is a better predictor of their creativity?

## Review of the Literature

### Grit

Grit is presented as a different characteristic obvious in successful education (Duckworth, 2016), and it is a notion that needs to be considered in terms of society and emotions and specific focus is placed on it regarding one’s achievement (Brooks and Seipel, 2018). Grit is considered as the capability to undergo hardships while maintaining wishes for long-run objectives (Eskreis-Winkler et al., 2014; Howard et al., 2019). As a composite and consistent personal feature characterization, grit affects mentalities and activities within diverse environments and is common among the unique pioneers in each area (Wolters and Hussain, 2015). In addition, grit can be described as the passion and decision to obtain long-haul goals, regardless of problems and challenges, and it may be lively personality power for the conditions when individuals feel their personal concerns and problems or when they experience vital conditions (Lozano-Jiménez et al., 2021). Usually, grit has the potential to preserve passion and effort in works needing time for being completed. Those who do not avoid their main goals have a great grit degree. Therefore, the grit may be naturally considered follow-thru or the deliberate, ongoing dedication to practices and responsibilities experienced to perform one’s goals efficaciously (Duckworth and Quinn, 2009; Tough, 2012). Therefore, the core of grit can be viewed as the follow-through or the deliberate, consistent commitment to practices and accountabilities encountered when trying to attain one’s goals efficaciously (Duckworth et al., 2007). Research about grittier individuals indicated an improvement in educational and non-educational performance and increased motivation as they understood meaning in achieving success (Von Culin et al., 2014). An effective individual with high persistence is both greatly inspired and keen on focusing on attaining long-run, more aspiring objectives and is flexible and less attentive to day-to-day routines (Duckworth, 2016).

Grit is the ability to pay attention to and defeat problems while also keeping the grit to fight with difficulties (Duckworth et al., 2007). The grit incorporates mechanisms at the trait level of persistence and enthusiasm for long-term objectives (Duckworth and Quinn, 2009; Duckworth, 2016). The persistence of attempt alludes to keen preference and grit to achieve a purpose and pertaining grit with resiliency, purposefulness, decision, and precision, and it describes the extent to which people may tolerate obstacles and hardships as helping the strange power (Duckworth et al., 2007; Datu et al., 2019); however, the other one is the enthusiasm for long-run decisions which refers to individuals’ ability to focus for an extended length which also alludes to the degree to which people commonly emphasize completing their continued objectives (Datu et al., 2019).

Gritty human beings show advantages in learning achievement, the continuation of involvement, and persistence thru difficulty in education (Eskreis-Winkler et al., 2014). On behalf of researchers, many signals exist confirming the important relationship between grit and constructive outcomes (Eskreis-Winkler et al., 2014). As an example, people with greater grit obtain significantly higher scores, both in school and at university, and continue completing more creative levels of knowing about those who display low levels of grit whereas higher levels of grit specify better achievement in after class responsibilities (Duckworth and Quinn, 2009) and enjoy significantly higher levels of school fulfillment (Eskreis-Winkler et al., 2014). Therefore, Duckworth et al. (2007) built a 12-item Grit Scale and determined two lower-order constituent concepts: (1) uniformity of interest, characterized as a person’s long-term fervor for a long-run objective despite problems, impediments, setbacks, frustration, or difficulties they might face, and (2) persistence of endeavor, which alludes to a person’s liking to invest non-stop attempts when seeking a long-run objective. Duckworth and Quinn (2009) then built and proved a short grit scale incorporating eight items estimating the same two elements.

Moreover, grit is defined as a primary and inevitable matter in achieving societal-expressive development in all other dimensions of life and can be regarded as a societal-expressive skill or ethical value (Sarıçam et al., 2016). Investigations have demonstrated that grit is linked to achievement, self-effectiveness, self-control, metacognition, hopelessness, and worry (Wolters and Hussain, 2015; Lee, 2020). Grit includes engagement in a superordinate objective that is positioned at the peak of a pyramid while base goals are strongly related and intend to move in the path of the superordinate objective (Duckworth and Gross, 2014).

### Resilience

Resilience pertains to protective and vulnerable factors inside and outside people which affect people’s regulation of alterations and disturbing experiences that lead to a loss of homeostasis (Brewer et al., 2019). It is pinpointed that a resilient person is described based on their capability to cope with alterations. Consequently, Portnoy et al. (2018) believe that resilience is related to how individuals can get better when facing a difficult situation or coping with it. The resilience procedure focuses on the relationship between people and their environment and assesses the association between different dimensions of psychological well-being and educational fulfillment (Van der Meulen et al., 2020). Besides being able to conform to challenging conditions, resilience may be comprehended as the strength to adjust to the modifications in conditions (Southwick et al., 2014). That is, resilience is the potential to respond in a self-assured way to difficulty or adversity in a certain condition (Masten and Reed, 2002).

A resilient individual grows the potential to handle the mistakes and setbacks instead of permitting negative conditions to impede their function. Educational achievement requires specific cognitive skills. Enjoying resilience means the ability to stay alive and handle the difficult effects of stressful conditions and different difficulties faced regularly (Richardson, 2002). In the education profession, resilience is a lively necessity to understand education and academic methods that take place whilst individuals relate their assets with subject-centered ones and employ operative methods and strategies to defeat problems and maintain their well-being (Greenier et al., 2021). Resilience is a lively and multidimensional notion, where the relations of individual and related sources can develop (Beltman and Mansfield, 2018; Gu, 2018). Similarly, resilience, as the skill of handling hardships and problems swiftly and successfully, has been demonstrated to be in a negative relationship with worry, sadness, and anxiety (Connor and Davidson, 2003), and a positive relationship with the feeling of joy and an individual’s health (Fredrickson, 2001).

### Creativity

To characterize creativity, Piirto (2004) draws attention to the origin of the words “create” and “creativity” which mean “to generate or build.” As for creativity, scholars concur that creativity alludes to the creation of notions or products that are innovative, of value, or beneficial. Creativity is a mental concept that experts and non-experts appear to comprehend but have a difficult time characterizing. This could be due to its overlap with conventional personal contrast classes (Dörnyei, 2005); for example, it is one of the three fundamental dimensions of Sternberg’s theory of efficacious intelligence (Sternberg, 2017). In the academic area, creativity instruction incorporates the growth of a mixture of skills, abilities, demeanors, inspiration, information, and other traits (Starko, 2013). Creativity is characterized as the cycle by which individuals contemplate and act to create new works. Creativity is said to incorporate four stages: arrangement, incubation, clarification, and confirmation (Xu et al., 2021). After tens of years of comprehensive exploration, a more profound comprehension has been built regarding the capabilities, attributes, and intellectual cycles related to creativity, and scholars have adopted a more subtle and interdisciplinary method for comprehending creativity (Cadle, 2015). For instance, new grounded-theory research suggested that the creative cycle for journalists involves four steps: intellect, improvement, capture, and contemplation (Rubenstein et al., 2013). Educator’s creativity is a vital element of entrepreneurship education with the result of learning severe entrepreneurship. The creativity of this educator ought to continue advancement so that entrepreneurship education efficaciously influences the goals of entrepreneurial learners (Beghetto and Kaufman, 2014). Moreover, creativity is essentially required for two vital purposes: Firstly, modern communities hopelessly want the creative feature of enhancing their enterprises (Beghetto and Kaufman, 2014; Wibowo and Saptono, 2018). Alenizi (2008) stipulated that the features within creativity, namely, resilience, validity, compatibility, and validity are essential for life requirements as well as for the business sustainability. Second, the educators’ creativity leading to learners’ creativity considerably helps the fulfillment of learning in the class.

## Materials and Methods

### Participants

To accumulate the data, a convenient sample of 264 participants from 11 provinces (Fujian, Liaoning, Guangdong, Shandong, Jilin, Zhejiang, Anhui, Sichuan, and Hunan) and two municipalities directly governed by the central government of China (Shanghai and Chongqing) was recruited. To generalize the research samples, the research participants comprised both male (N = 110) and female (N = 154) Chinese EFL teachers at Master’s and Ph.D. levels, whose majors included English literature, theoretical linguistics, business English, and English translation studies, language education, and others. Their age ranged from 23 to 56, with an average age of 35.

### Instruments

#### Grit Scale Questionnaire

The scholar employed the Grit-S as a roughly brief Likert scale eight-item inventory which has two scales, such as stability of interest and persistence of attempts. Such scale was designed and confirmed by Duckworth and Quinn (2009) named the Grit Scale inventory wherein items are graded on a five-point scale from (1 = never like me to 5 = very similar to me) to assess how gritty an individual is. It is worth noting that the reliability of the questionnaire was calculated through Cronbach’s α value and it was.824.

#### Connor-Davidson Resilience Scale

Connor and Davidson (2003) conducted a study in which they changed the scale of educators’ degree of resilience. Even though the authentic CD-RISC consists of 25 items with five dimensions parallel to the foundations of the notion, several items in the authentic scale might not be used in the sample. Thus, according to Connor and Davidson (2003), only ten objects were chosen for the present scale equal to the primary three problems produced from the authentic scale, which include “the concept of individual skill, great criteria, and difficulty,” “reliance on an individuals’ instincts, bearing negative effect, and reinforcing impacts of tension,” and “constructive admittance of modification, and safe relations” (Connor and Davidson, 2003). Such items need to be replied to with a 5-point Likert scale ranging from 0 (not true at all) to 4 (true nearly all the time). The internal consistency of the scale was 0.858 calculated by running Cronbach’s α value.

#### Creativity Scale

The scholars utilized the creativity scale as a relatively 47-item and 4-point Likert survey involving four subscales (strongly disagree to strongly agree) including educator self-effectiveness, surrounding motivation, social worth, and educator capacity. This scale was created and confirmed by Rubenstein et al. (2013). The reliability of the scale was 0.835 calculated by running Cronbach’s α value.

### Procedure

In the first place, an informed consent form was signed by all those participants to indicate their willingness to participate in the current study. For the convenience and administration of the data collection during the period of the COVID-19 pandemic, all the questionnaires were translated into Chinese, well prepared online, and distributed *via* WeChat in the form of internet linkage. It took the participants around 20 min to fill out the questionnaires. In addition, to collect the trustworthy data, the researcher of the present study distributed the questionnaire to different provinces to get the relatively large-scale answers. This data collection lasted for 1 month, beginning on January 5th, and ending on February 16th.

### Data Analysis

In agreement with the objective of the study, to answer the first research question of the study, correlation analysis was used, while for the third question, a linear multiple regression was run.

## Results

A detailed analysis of the data associated with creativity, grit, and resilience is provided focusing on how creativity is related to grit and resilience. As the first step of the data analysis, the reliability of the scales was estimated. Table 1 indicates the results of the reliability for the three scales of the study.

**TABLE 1:** Reliability statistics of the scales.

	Cronbach’s alpha	Cronbach’s alpha based on standardized items	No of items
Resilience	0.858	0.859	10
Creativity	0.835	0.865	43
Grit	0.824	0.539	8

As presented in Table 1, the resilience and creativity scales had reliability scales higher than 0.70, which indicates the two scales have adequate reliability (De Vellis, 2003). As for the grit scale, Cronbach’s Alpha was 0.48, which was lower than 0.70. However, the grit scale has only eight items which is less than the recommended number of 10 (Pallant, 2010). In such cases, it is suggested that mean inter-item reliability is reported (Pallant, 2010). According to Briggs and Cheek (1986), any number within the range of 0.2 to 0.4 is considered a good index of mean inter-item correlation. The average of the item correlations was computed and was found to be 0.25. Hence, all three scales had adequate reliability, and obtained scores were reliable.

In this study, 264 EFL teachers participated by completing three scales measuring creativity, grit, and resilience. Table 2 displays the statistics of the teachers in resilience, grit, and creativity.

**TABLE 2:** Statistics of the participants about resilience, grit, and creativity.

		Statistic							Std. error		
	N	Mean	Median	Std. deviation	Minimum	Maximum	Skewness	Kurtosis	Mean	Skewness	Kurtosis
Grit	264	18.07	17.00	3.891	10.00	31.00	0.773	0.306	0.239	0.150	0.299
Resilience	264	36.98	38.00	6.276	10.00	49.00	−1.132	2.135	0.386	0.150	0.299
Creativity	264	131.59	133.0	11.99	43.00	170.00	−2.065	12.154	0.738	0.150	0.299

**FIGURE 1:**
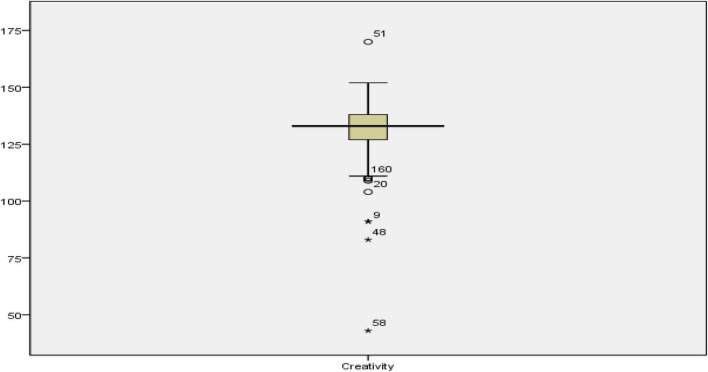
The boxplot for creativity scores.

**FIGURE 2:**
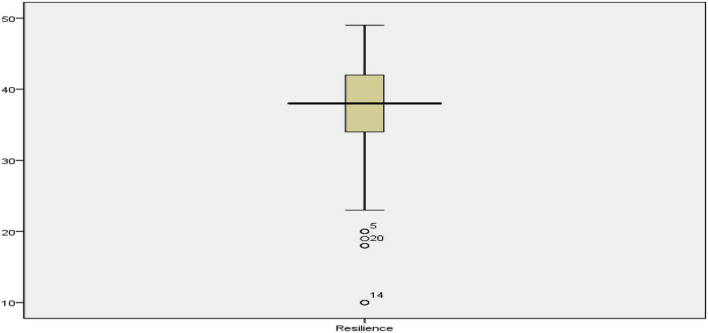
The boxplot for resilience scores.

**FIGURE 3:**
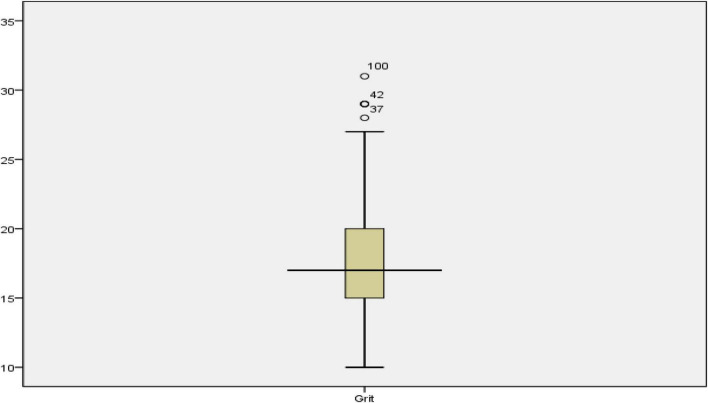
The boxplot for grit scores.

As presented in Table 2, the mean scores (std. deviation) of the participants in grit, resilience, and creativity were 18.07 (*SD* = 3.89), 36.98 (*SD* = 6.27), and 131.59 (*SD* = 11.99), respectively. Before starting any further analysis, the outliers in the data were examined as they could affect many of the assumptions of the multiple regression for addressing the research questions. Figures 1–3 show the boxplots for identifying the outliers in creativity, resilience, and grit scores.

As indicated in Figures 1–3, the cases 51, 160, 20, 9, 98, and 58 in creativity scores, 5, 20, and 14 in resilience scores, and 100, 42, and 37 in grit scores were outliers. All these outliers were removed from the analysis before addressing the research questions. Table 3 shows the descriptive statistics after removing the outliers.

**TABLE 3:** Statistics of the participants with regard to resilience, grit, and creativity after removing the outliers.

		Statistic							Std. error		
	N	Mean	Median	Std. deviation	Minimum	Maximum	Skewness	Kurtosis	Mean	Skewness	Kurtosis
Grit	253	17.82	17.00	3.64	10.00	29.00	0.690	0.167	0.229	0.153	0.305
Resilience	253	36.98	38.00	6.27	10.00	49.00	−1.132	2.135	0.386	0.150	0.299
Creativity	253	132.23	133.00	9.94	83.00	152.00	−0.935	2.682	0.625	0.153	0.305

As presented in Table 3, the mean scores of the participants in grit, resilience, and creativity were 17.82 (*SD* = 3.64), 36.98 (*SD* = 6.27), and 132.23 (*SD* = 9.94), respectively. The distribution of the data of the variables seems to be normal as the mean scores of resilience, well-being, and grit were close to their median. Normality of the data was also confirmed *via* the Normal Probability Plot (Pallant, 2010) which is explained when addressing the assumptions of the multiple regression in the following sections.

**FIGURE 4:**
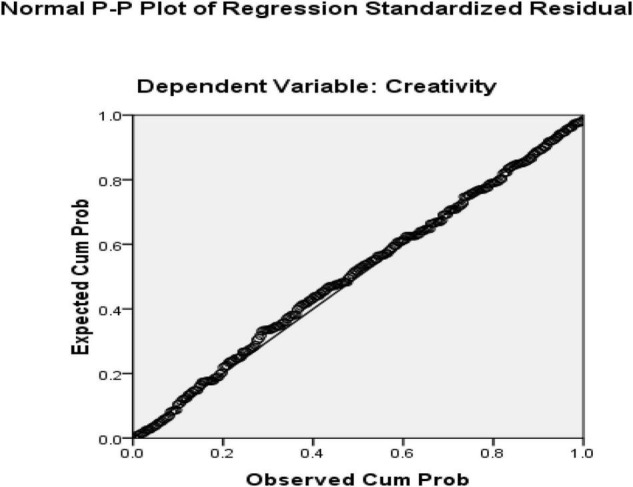
Normal Probability Plot (p-p) of the Regression Standardized Residuals (independent variable = resilience and dependent variable = creativity).

**FIGURE 5:**
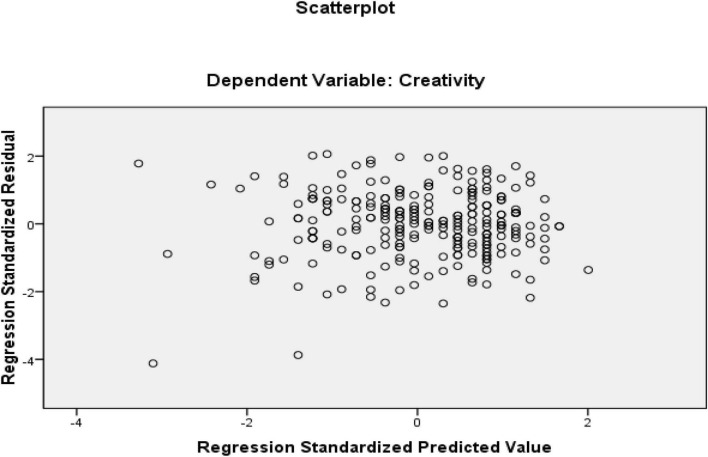
Scatter plot of residuals (independent variable = resilience and dependent variable = creativity).

The first research question was about how resilience predicts creativity. The answer to this question was sought using a linear multiple regression. Creativity was defined as the dependent variable in the multiple regression model while resilience served as the predictor. Before performing regression analysis, certain assumptions needed to be met. These assumptions require that variables are continuous, the sample size is large, there is no multicollinearity, there are no outliers, data is normally distributed, relationships between predictors and dependent variables are linear, variances of residuals are the same (homoscedasticity), and residuals are independent (Pallant, 2010). All the variables of the study were continuous, ensuring the variables’ continuous nature. Additionally, all outliers were removed from the present research at the beginning of the data analysis as for the sample size which sample in this study included 253 EFL teachers, which is satisfactory for multiple regression. The multicollinearity assumption requires that the predictors do not have a strong relationship. Since we only entered one predictor (resilience) in our regression model, this assumption is automatically satisfied. Normal Probability Plot (P-P) of the Regression Standardized Residuals and the Scatterplot were consulted to check the assumptions (Figures 4, 5).

Based on the normal probability plot, all the points indicate the data’s normality. Additionally, residuals in the scatter plot are centralized in a rectangular form with no apparent pattern that indicates linearity, homoscedasticity, and independence of residuals (Pallant, 2010). In the next step, the regression output was consulted to check how resilience predicts creativity (Table 4). In the regression output, the R Square value multiplied by 100 indicates the prediction power of the model. The ANOVA table (Table 5) shows the significance of the regression model.

**TABLE 4:** Regression model output.

Model	R	R square	Adjusted R square	Std. error of the estimate
1	0.334^a^	0.112	0.108	9.417

*^a^Predictors: (Constant), Resilience.*

**TABLE 5:** ANOVA for regression.

Model		Sum of squares	df	Mean square	F	Sig.
1	Regression	2829.915	1	2829.91	31.90	0.000*^a^*
	Residual	22528.71	254	88.696		
	Total	25358.62	255			

*^a^Predictors: (Constant), Resilience.*

**FIGURE 6:**
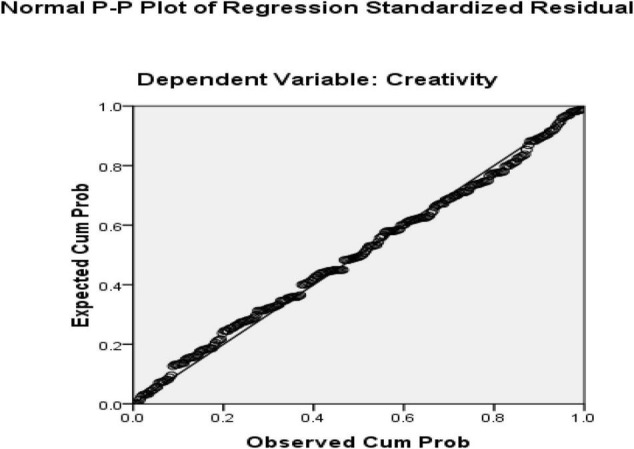
Normal Probability Plot (p-p) of the Regression Standardized Residuals (independent variable = grit and dependent variable = creativity).

**FIGURE 7:**
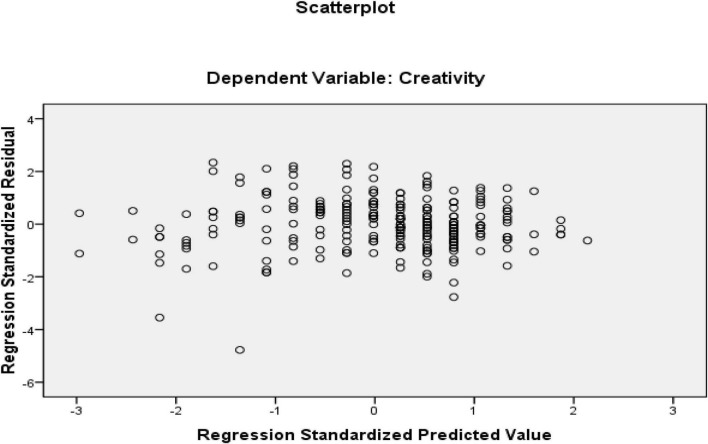
Scatter plot of residuals (independent variable = grit and dependent variable = creativity).

Based on regression and ANOVA output, resilience could predict 11% of the variance (R Square = 0.112) in creativity which was significant (*f* = 31.90, *P* = 0.00). The second research question was about how grit predicts creativity. The same analysis performed for the first research question was repeated for the second research question. Therefore, the multiple regression assumptions were first checked, and then the regression output was consulted to find the answer to the second research question. All the variables of the study were continuous, which ensures the continuous nature of the variables. Additionally, all outliers were removed from the study at the beginning of the data analysis. The sample included 253 EFL teachers, which is beyond the recommended sample size by Tabachnick and Fidell (2007). The multicollinearity assumption was already met as there was only one predictor (grit). Normal Probability Plot (P-P) of the Regression Standardized Residuals and the Scatterplot were consulted to check the required assumptions (see Figures 6, 7).

Based on the normal probability plot, all the points indicate the data’s normality. Additionally, residuals in the scatter plot are centralized in a rectangular form with no significant pattern that indicates linearity, homoscedasticity, and independence of residuals (Pallant, 2010). After multiple regression assumptions were ensured, the main regression output and ANOVA output were examined (Tables 6, 7).

**TABLE 6:** Regression model output.

Model	R	R square	Adjusted R square	Std. error of the estimate
1	0.402*^a^*	0.162	0.158	9.14907

*^a^Predictors: (Constant), Grit.*

**TABLE 7:** ANOVA for regression.

Model		Sum of squares	df	Mean square	F	Sig.
1	Regression	4081.716	1	4081.716	48.763	0.000^a^
	Residual	21177.467	253	83.705		
	Total	25259.183	254			

*^a^Predictors: (Constant), Grit.*

Based on regression and ANOVA output, resilience could predict 16% of the variance (R Square = 0.162) in creativity which was significant (*f* = 48.76, *P* = 0.00). The third research question aimed to detect which predictor (grit or resilience) is a better predictor of creativity in EFL teachers. As evident in previous sections, resilience could predict 11% of the variance in creativity, and grit could predict 16% of the variance in creativity. In other words, the regression results suggest that grit compared to resilience is a better predictor of creativity in EFL teachers. To further analyze how grit and resilience are related to creativity, the correlation between grit and creativity and between resilience and creativity were examined. Table 8 shows the correlations between creativity and the predictors (grit or resilience).

**TABLE 8:** Correlations between creativity and the predictors.

		Grit	Resilience
Creativity	Pearson Correlation	−0.402**	0.334**
	Sig. (2-tailed)	0.000	0.000
	N	255	256

**Correlation is significant at the 0.01 level (2-tailed).

Based on the correlation coefficients, creativity was negatively but significantly related to grit (*r* = −0.40, *P* = 0.00), but it was positively and significantly related to resilience (*r* = 0.33, *P* = 0.00). Therefore, although the relationship between grit and creativity is negative, it is stronger than the relationship between resilience and creativity.

## Discussion

This study inspected the role of grit and resilience on teachers’ creativity in language education. To this end, the grit, resilience, and creativity of 253 Chinese EFL teachers were measured using questionnaires. The obtained scores were evaluated using multiple regression and correlation analysis. The results of the multiple regression showed that both grit and resilience could significantly predict creativity. It was also shown that grit is a better predictor of creativity. Correlation analysis showed that the correlation between grit and resilience is negative but is stronger than the association between resilience and creativity. The findings indicate that grit and resilience both were predictors of teachers’ creativity. Hypothetically, these upshots support a developing view of the function that PP can have in the educational process (Wang et al., 2021). It can be concluded that positive emotions make people more creative and even make the creative progression easier (St-Louis and Vallerand, 2015). The results support those of other research studies in which they proved the positive correlation between creativity and resilience among Chinese (Seale et al., 2013; Chen et al., 2018). Creativity refers to the capability to introduce something new and considering that people are unable to create something out of nothing, their creation constantly includes reforming the provided content, either physical or psychological. Such description significantly echoes with those of resiliency, mainly described as bouncing back thru positive compatibility – a reforming caused by a provided condition like difficulty or danger (Masten and Powell, 2003). By presenting the possible positive relationship between resilience and creativity among teachers in China, we expand the existing comprehension in the academic literature that these teachers’ positive mental condition as resilience is vital for efficient creative working. Resilient people can be away from stressful incentives when they are in the face of adversities and they usually try to find something interesting that helps them be involved in an activity that just motivates them, without expecting any rewards for it. Regarding the role of resilience and creativity, the results proved the relationship between these two constructs that it is concluded that creativity can specifically affect the language procedure which is in line with the notion of broaden-and-build theory (Fredrickson, 2001). A potential clarification is that positive affections may change a language learner’s orderly modes of thought and lead to higher levels of creative and resilient modes of thought. Therefore, thru such a broad-minded agenda with a broad scope of creative behavioral alternatives and abnormal patterns of thinking, which are provoked by positive affections, one can enhance the potential methods by which language teachers can deal with the difficulties faced when involved in education (Fredrickson et al., 2003). The results are consistent with the study by Seale et al. (2013) and Chen et al. (2018) who declared that creativity is an individual attribute and in a positive association with resilience. It is contended that those who are creative contemplators are prone to not accept losing hope when encountering hardships or difficulties; rather, they look for substitute ways and proceed to pose “what if” inquiries (Kaufman, 2016).

In addition, among the constructive affections related to the creative procedures along with the wonder and surprise, enthusiasm, as a secondary element of grit is also chosen, as having an exploratory feature, assessment, and is a dynamic pressure (Tokarz, 2005). The results on the grit-creativity association advocate the prior work conducted by Fredrickson (2001) indicating that people with greater points on positive affections are inclined to have greater degrees of creative and resilient intellect. Baas et al. (2008) in their meta-analysis of 25 years of the state-creativity literature indicated that constructive states have the possibility of triggering people’s creative degree more than a neutral state. Davis (2009) found identical findings, indicating that a positive state can enhance creativity after reviewing 72 chosen studies. The findings are in line with Grohman et al. (2017) and Rojas and Tyler (2018), who determined that grit affects instructing creativity, signaling its importance for institutional devotion, information administration, and creativity among educators in Indonesia. Grit in instructing creativity is crucial and the inclination to sustain devotion, concentration, and attempts to endeavor for the attainment of objectives/assignments of education as a long-run grit indicator is required by educators to develop instructing creativity, either directly or indirectly moderated by institutional devotion and information administration. When it comes to explaining grit’s connection to creativity, it can be inferred that it is uncommon for people to practice creativity in areas that are not interesting to them or for which they do not sense any individual relationship—except maybe when trying to understand how they can evade those domains (Tomlinson, 2013). The grit indices, namely, stability of favorites and perseverance of attempts (Duckworth and Quinn, 2009), if adequate and continued in the long term, may provoke educators’ dedication, knowledge control, and education creativity. Concerning the creativity-grit relationship, creativity can strengthen resiliency in people by aiding them to discover new solutions to difficulties. In addition, it can help individuals in considering issues and difficulties from various viewpoints at the same time as letting them question and go past the autocratic customs that capture them (Metzl and Morrell, 2008).

## Conclusion and Implications

Educators with a great level of creativity are proficient in adjusting, explaining, and characterizing novel circumstances, and coming up with answers, including “chaotic” educational circumstances that rise without expectation in schools because of various troubles, demanding swift expectation and immediate response. Furthermore, educators with a great degree of instructing creativity will not have problems with difficulties, even when they are encountering novel issues in the instructing domain.

The results of the study are significant for teacher trainers as the grit of teachers needs to be enhanced uninterruptedly through employing the appropriate strategy. Indeed, educators require to autonomously and purposely enhance their grit potential through reading diverse related literature since grit is concerning the teachers’ creativity. Moreover, school heads need to start and aid educators’ engagement in education programs designed in particular to enhance grit. The education programs have to engage professional educators who are completely skillful in the grit area. The provided content needs to improve stability of interests and endurance of attempt for achieving tutorial academic aims thru educational creativity, and the approaches must be employed based on the wishes of the education content, integrating talking and debates, namely, discussions of concentration group, provocations, and playing roles. The training plans should include professional educators who are highly capable in the domain of grit. The content offered must improve the constancy of interests and perseverance of endeavor for the achievement of academic objectives through teaching creativity, and the approaches must be utilized based on the requirements of the coaching content.

In addition, based on the results, there are multiple practical applications for teachers. Indeed, the present study has implications wherein educators, with the help of school beneficiaries, mainly school heads, attempt to enhance their grit stability by using accessible school assets. Moreover, other academic institutions, which include colleges, public institutions (governmental), social institutions, and commercial institutions may use the findings of this study, mainly for the advantage of manpower improvement. First, also, constructing resilience can assist learners to discover creative methods for coping with their specific challenges and problems. Considering the important function of resilience in gaining creativity, learners should be motivated to grow their internal elements of resilience, like optimism and resilience. Creative individuals regularly find methods to attend to an issue that others are incapable of seeing while possessing the skill to conquer impediments where others might otherwise lose hope. With greater degrees of resilience, people are better capable of directing hardships and issues, which in turn, affects degrees of independence, regulation, and creativity, contributing to health, as well.

Since the population of the current study was from colleges in China, so the upshots may not be generalizable to other populations. Further surveys should be carried out on the relations between these constructs examined in this study across several kinds of cultures, or age groups. In addition, it is recommended to carry out more research in this field to examine other factors predicting creativity besides grit and resilience.

## Data Availability Statement

The raw data supporting the conclusions of this article will be made available by the authors, without undue reservation.

## Ethics Statement

The studies involving human participants were reviewed and approved by Fuzhou University Zhicheng College Research Committee. The patients/participants provided their written informed consent to participate in this study.

## Author Contributions

JS independently designed the current study, drafted the manuscript, and approved its submission to this journal.

## Conflict of Interest

The author declares that the research was conducted in the absence of any commercial or financial relationships that could be construed as a potential conflict of interest.

## Publisher’s Note

All claims expressed in this article are solely those of the authors and do not necessarily represent those of their affiliated organizations, or those of the publisher, the editors and the reviewers. Any product that may be evaluated in this article, or claim that may be made by its manufacturer, is not guaranteed or endorsed by the publisher.
